# Supplementary figures and images for: Functional Properties and Storage Stability of Astaxanthin-Loaded Polysaccharide/Gelatin Blend Films—A Comparative Study
Source: Polymers (Basel). 2022 Sep 24;14(19):4001. doi: 10.3390/polym14194001 (PMC9573679; doi:10.3390/polym14194001)

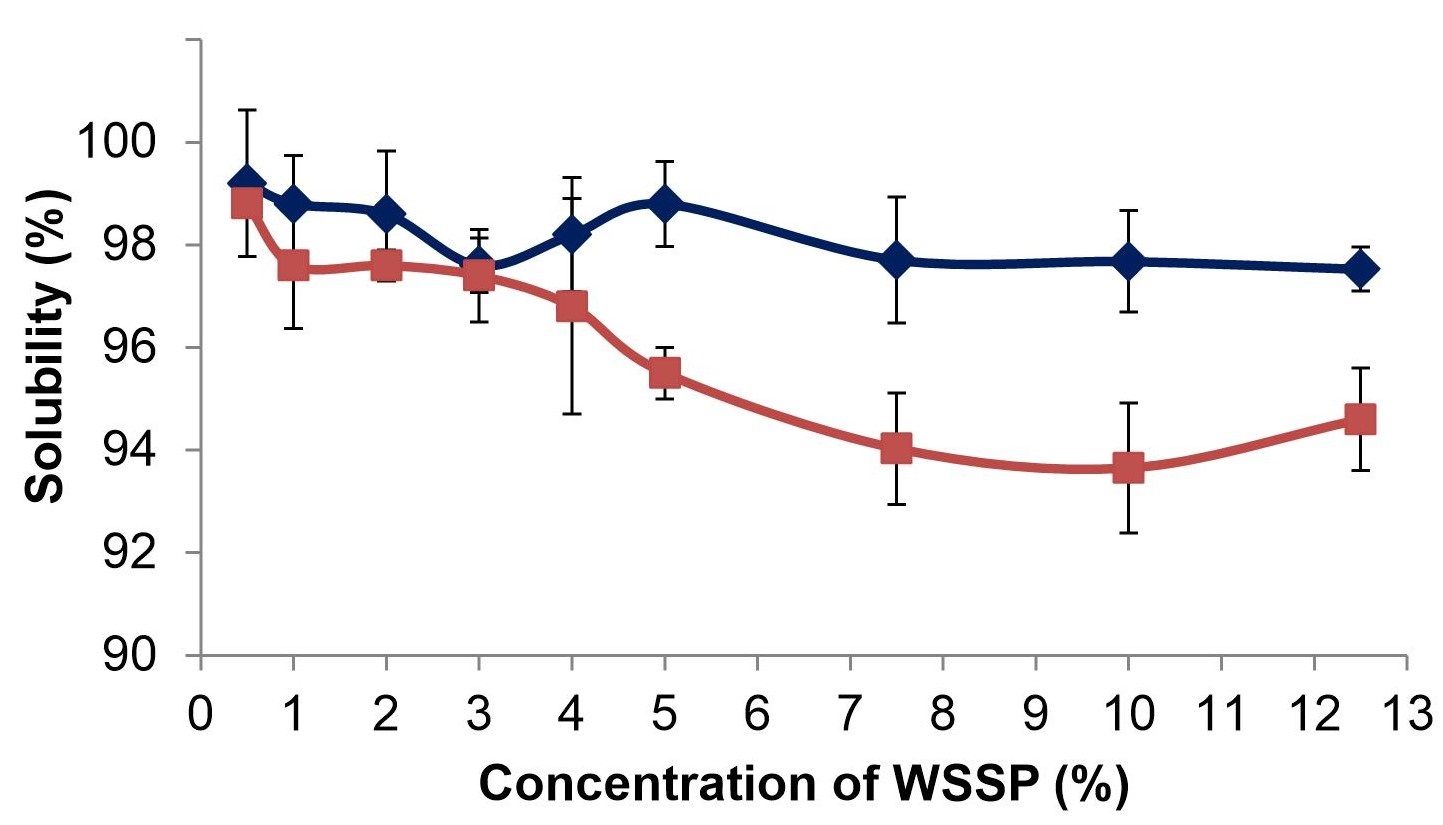

Supplement: Supplementary file 1 [file polymers-14-04001-s001.zip › Figure S1.jpg]

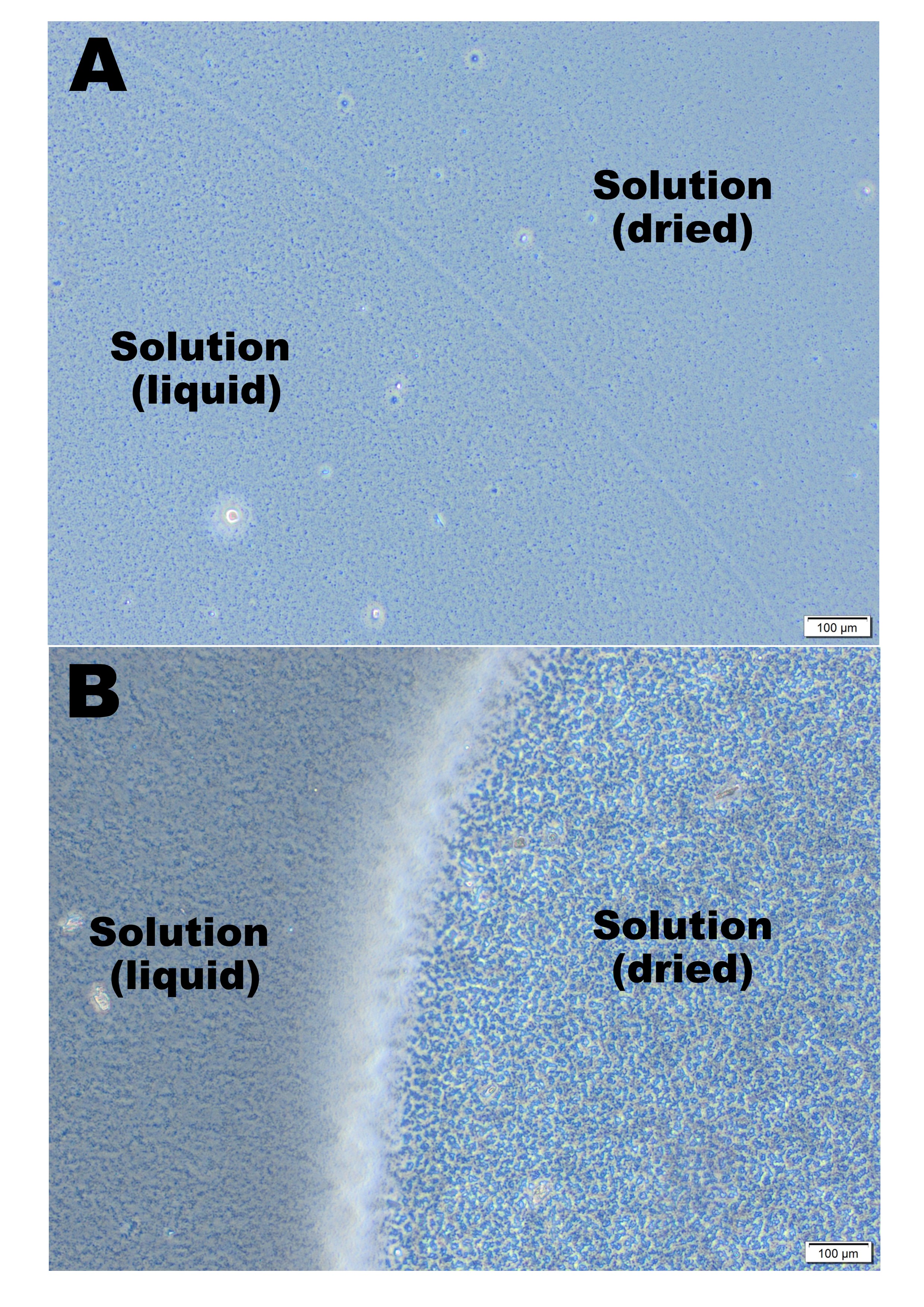

Supplement: Supplementary file 1 [file polymers-14-04001-s001.zip › Figure S2.jpg]

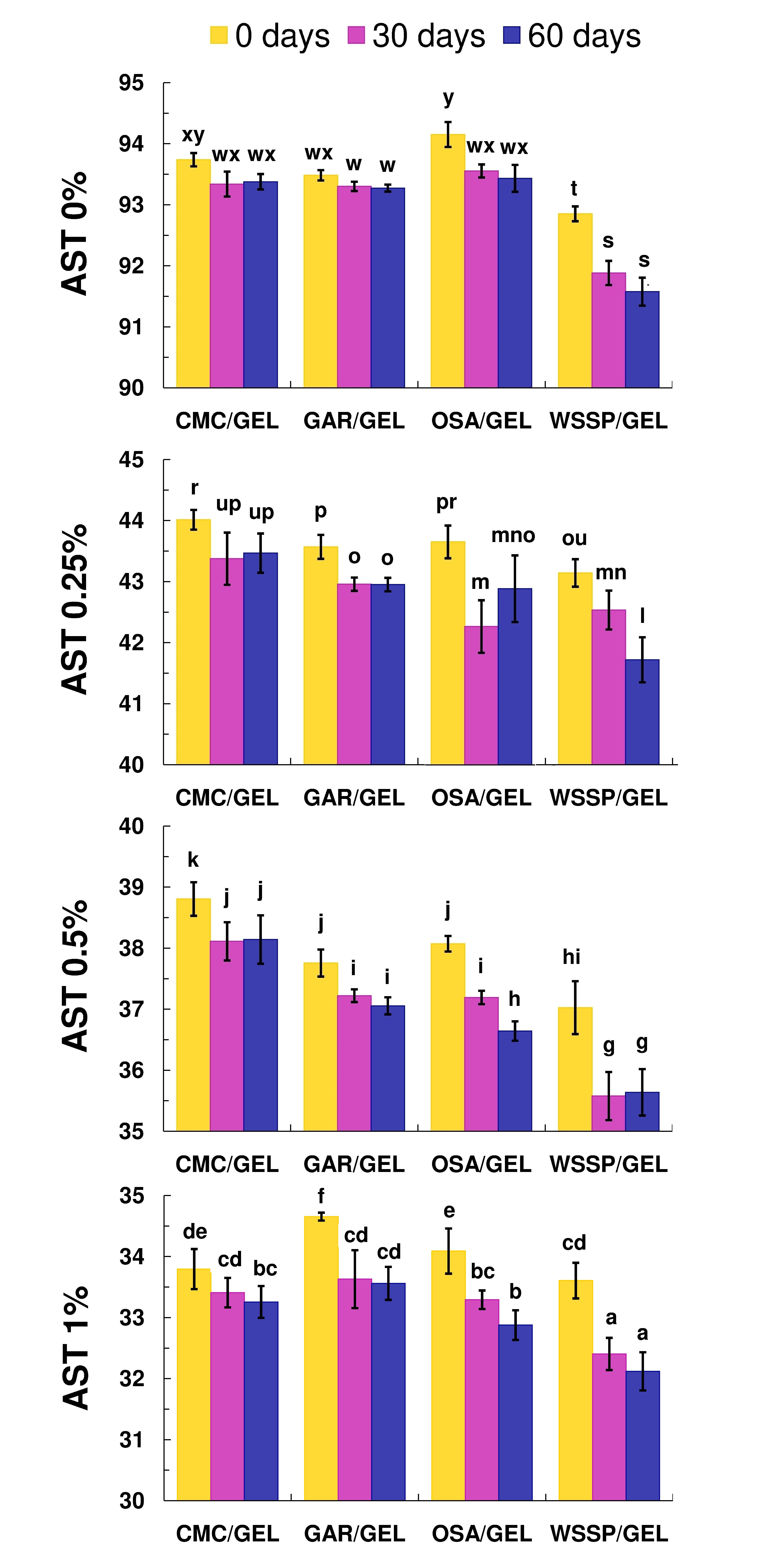

Supplement: Supplementary file 1 [file polymers-14-04001-s001.zip › Figure S3. L-60 days.jpg]

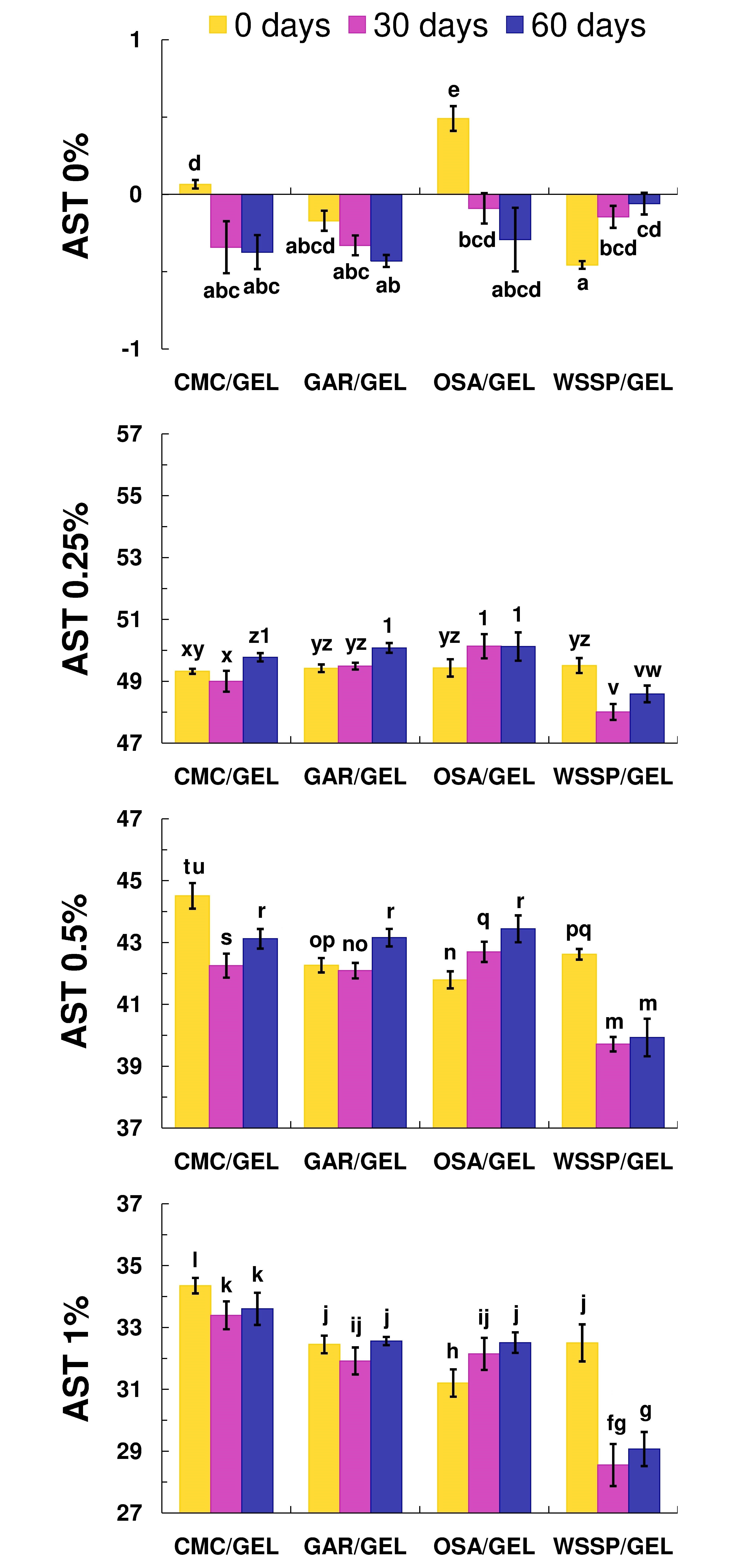

Supplement: Supplementary file 1 [file polymers-14-04001-s001.zip › Figure S4. a-60 days.jpg]

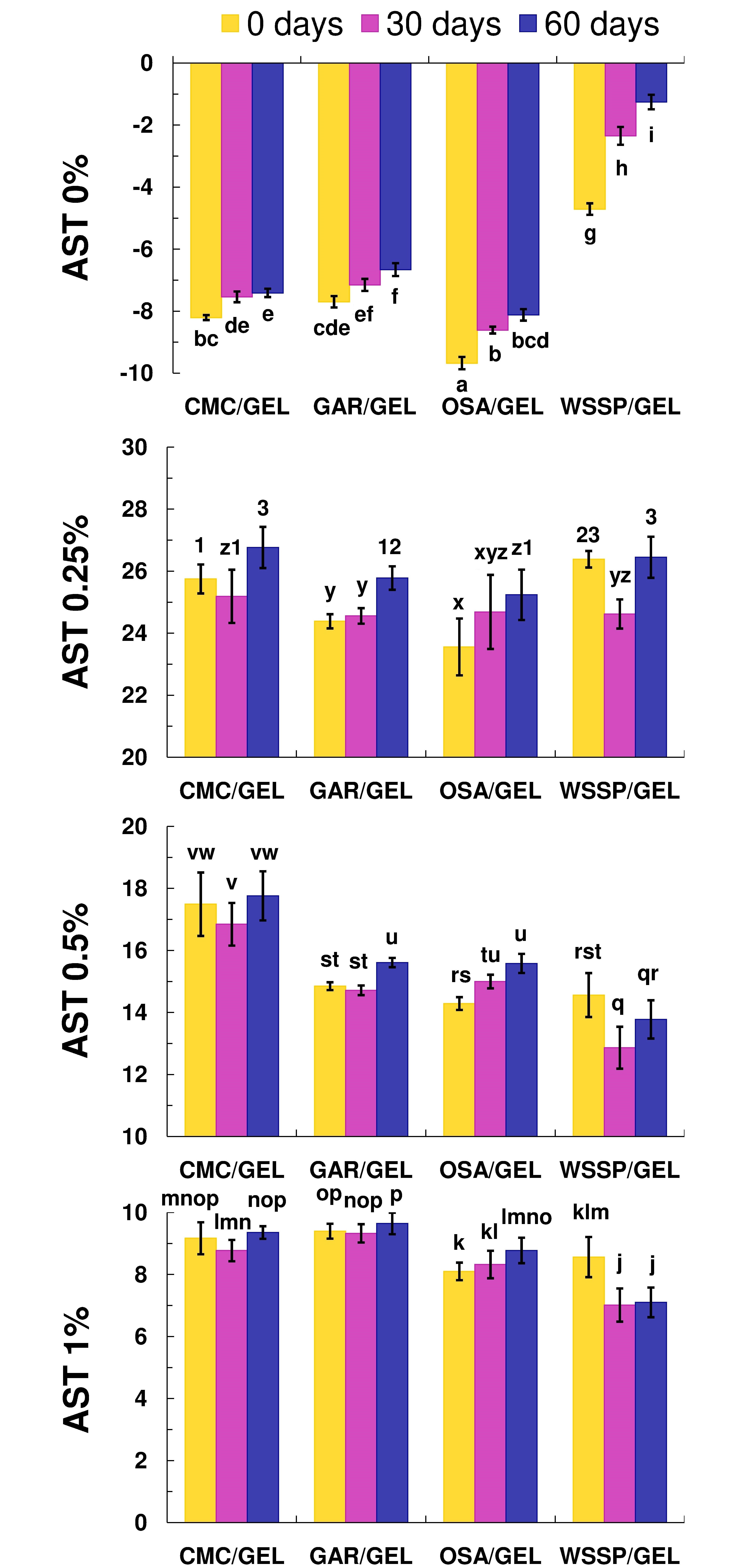

Supplement: Supplementary file 1 [file polymers-14-04001-s001.zip › Figure S5. b-60 days.jpg]

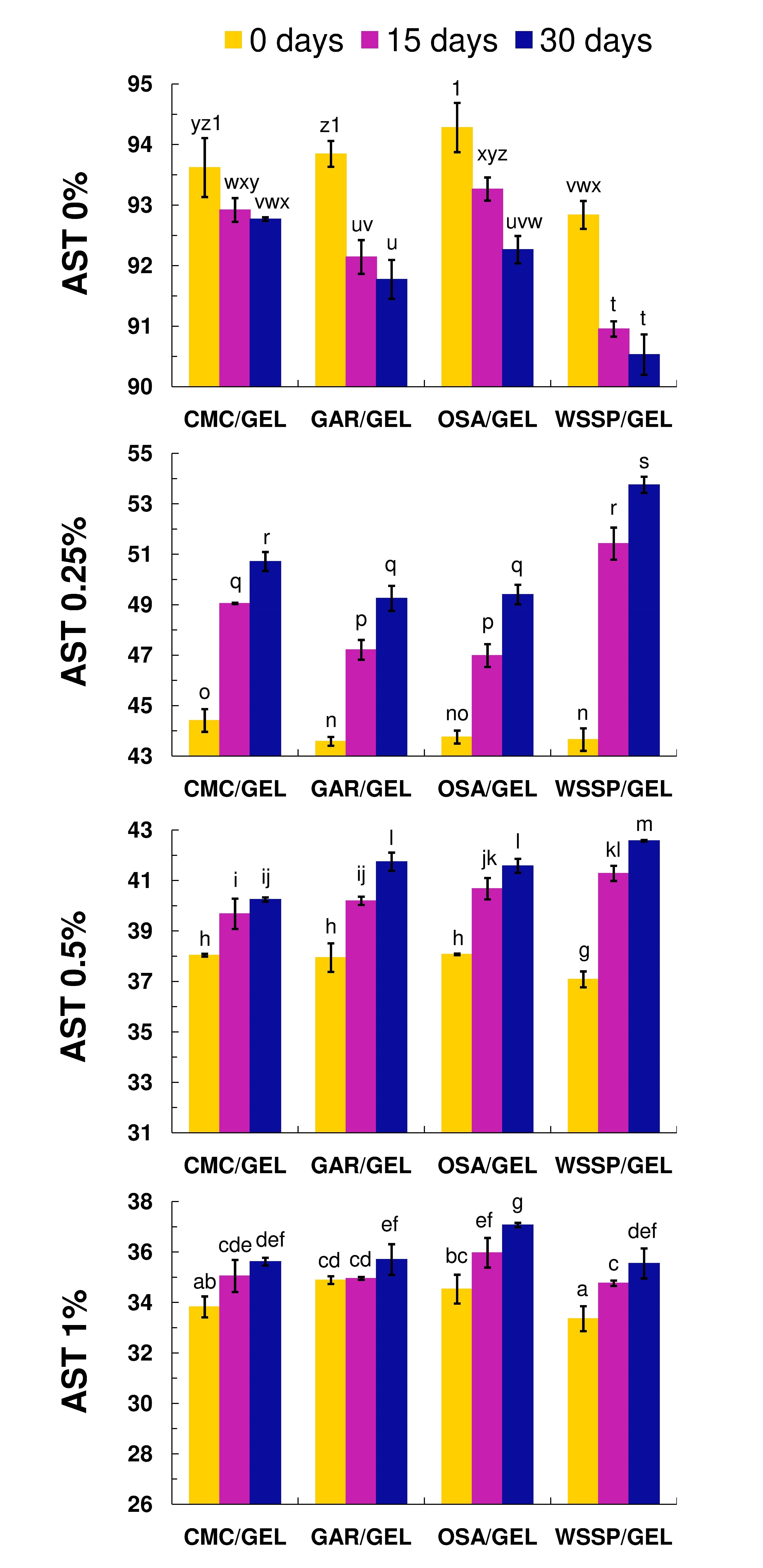

Supplement: Supplementary file 1 [file polymers-14-04001-s001.zip › Figure S6. L-30 days.jpg]

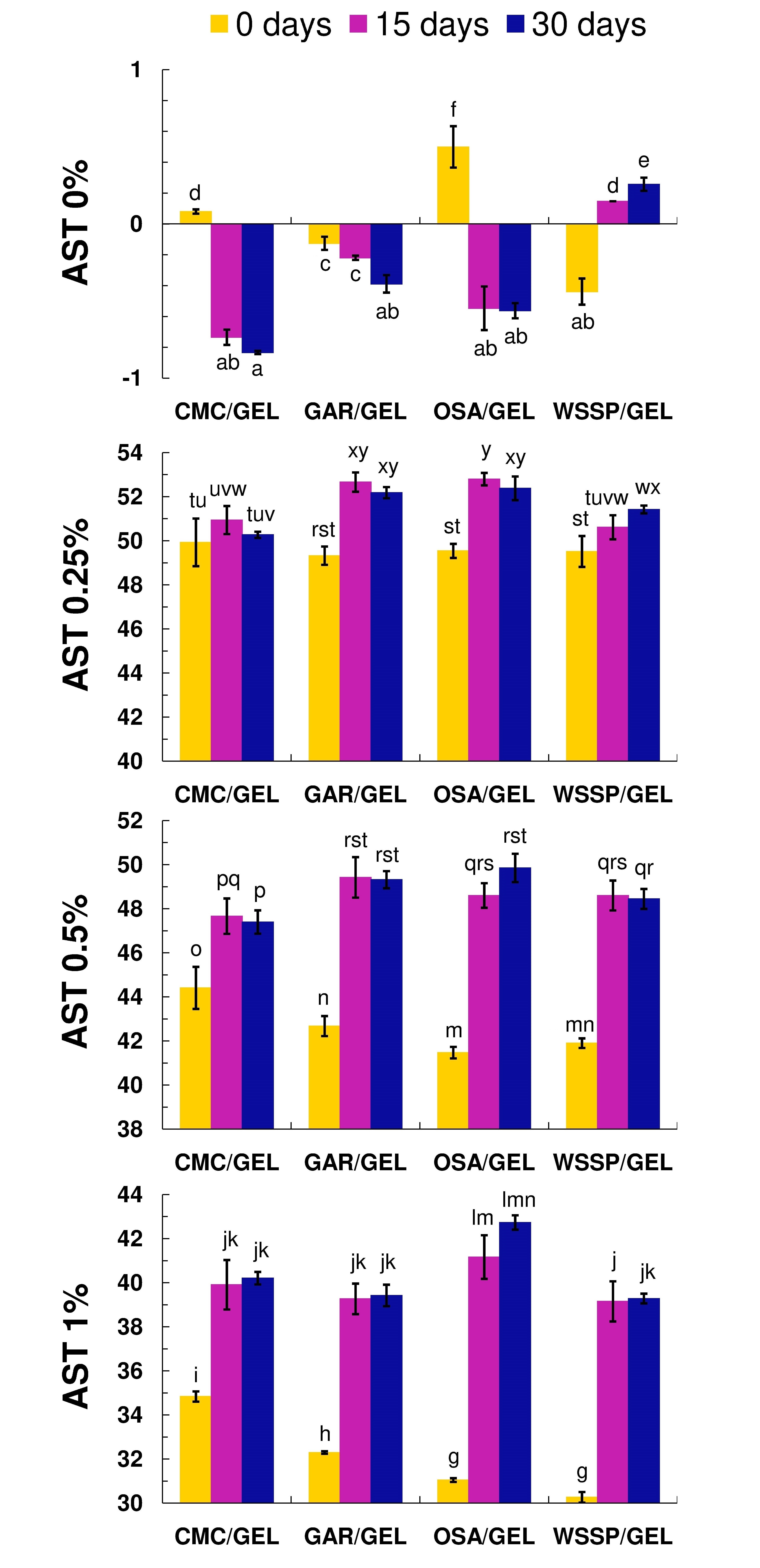

Supplement: Supplementary file 1 [file polymers-14-04001-s001.zip › Figure S7. a-30 days.jpg]

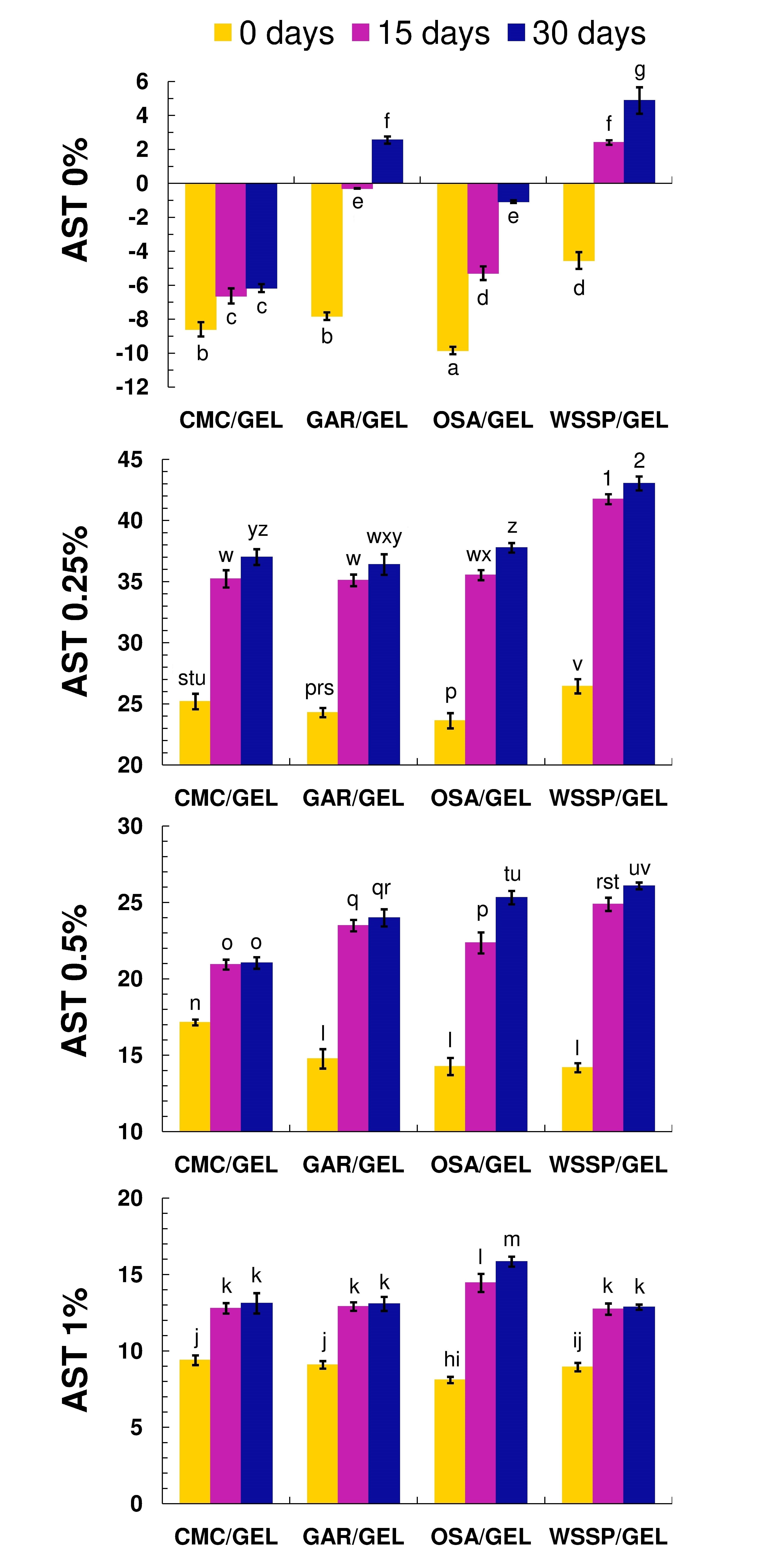

Supplement: Supplementary file 1 [file polymers-14-04001-s001.zip › Figure S8. b-30 days.jpg]
